# A high performance computing technology powered multimedia fusion model in university English translation

**DOI:** 10.7717/peerj-cs.1608

**Published:** 2023-10-06

**Authors:** Lin Shi, Minne DuJiang, Ping Gao

**Affiliations:** Xi’an University of Architecture and Technology, Xi’an, China

**Keywords:** Big data technology, Recurrent neural network, High performance computing, University English translation, Multimedia integration

## Abstract

Various forms of materials, such as pictures, videos and texts, have rapidly brought the college English translation teaching model into the era of multimedia integration. This makes it difficult for English teachers to improve college English translation by using unique materials, so as to form their own unique teaching style. In view of this, a multimedia comprehensive English translation framework based on the combination of big data technology and multimedia teaching mode is proposed. At the same time, the idea of building the framework is introduced from two perspectives: the integration of big data technology and multimedia, and the integration of multimedia and English teaching process. Then, a recursive neural network algorithm based on ant colony optimization algorithm is proposed and tested. Finally, the simulation results show that the proposed method has significantly improved the accuracy and retention rate, indicating the effectiveness of the framework.

## Introduction

There are large amounts of data collected from activities of daily life, governmental data analysis, market enterprise, and research entities, making big data appealing for its use in market applications (Zhou & Zhao, 2020). Market demand have promoted the popularization and application of big data technology (Jan et al., 2021). Big data is essentially the segmentation and processing of tasks, just like a computer’s data and processing capacity is relatively limited, the task is divided into multiple parts, data collection, integration, capture, and finally integration on each computer; now we are in a data resource pool, analyzing, mining, grasping and applying massive data to obtain useful data, thus helping to solve the problem. On the basis of big data, intelligent objects are continuously trained through logical algorithms, artificial neural networks (ANNs), and big data input samples to generate artificial intelligence. One of the key research areas in artificial intelligence is natural language processing (NLP).

Big data is scattered on the network in many forms, and it is a large field of collection of trillions of bytes of data, pet bytes, *etc*. Huge data clusters generated by a large number of users around the world exist mainly in the form of various unstructured big data (Momani, Abo-Hammour & Alsmadi, 2016). The huge data sets are imported into the memory for storage, and every moment the data is analyzed and categorized (Fadi & Deebak, 2020). The new technology operation is accompanied by a huge and complex data volume (Asch et al., 2018). In the face of this huge data base, the key to determining the recommendation quality of the recommendation system is the system’s data analysis and processing capability. The primary task of conducting big data analysis is to collect a large amount of data resources. Using IoT image recognition technology to collect reader data, the data source has the features of real-time, diversity and reliability (Za’er et al., 2013). The data research process, data information mining as the study of optimization algorithms based on the operation (Wang & Zhang, 2018). On this basis, the data information source is categorized and analyzed, and the operation law of the data itself is discovered in the analysis process, and then the design of the library recommendation system is realized to be applied in the reader recommendation service by relying on the optimized recommendation algorithm (Wang & Zhang, 2018; Shoumy et al., 2020; Zaer et al., 2014).

Teaching mode is a favorable teaching tool to assist teachers to complete teaching tasks, which is part of the teaching process and helps teachers to better achieve teaching objectives and teaching tasks (Shah et al., 2019). According to the teaching theory and teaching value orientation, the teaching mode is divided into many kinds, and the multimedia fusion mode is based on the theory of wisdom teaching, which is a new output result, and also an innovative result of the continuous development of the new curriculum teaching reform (Li, Huang & Emrich, 2019). The multimedia fusion mode is a new teaching ecological mode that introduces big data, the Internet and other modern information technology to realize teaching, which the teaching value orientation of the model is to realize the improvement of teaching quality by building a multimedia, intelligent, informative and digital teaching environment, and then improve students’ independent learning ability, their ability to solve learning problems, and continuously promote students’ wisdom production and development (Ma et al., 2018). With the pace of development of the new curriculum reform, social development has put forward higher requirements and standards for education and teaching, and the traditional teaching mode has been unable to meet the teaching requirements for education institutions (Li, 2021). In the background of big data, multimedia wisdom objectives include general objectives and wisdom teaching objectives, which are the basic contents of multimedia integration mode. Each small objective accomplished is the basis for the realization of multimedia wisdom objectives, which has a certain motivating effect on multimedia wisdom teaching activities (Qian, 2022).

With the proliferation of information resources and the increasing call from all walks of life to improve the level of English translation in universities, the difficulties of university teachers in organizing English translation materials become more and more difficult. These difficulties are mainly reflected in: (1) the information demand is time-sensitive and the demand changes rapidly; (2) the information demand is more extensive and presents systematic search; (3) the information demand tends to be diversified and is not only limited to image data such as figures, words and images, but also needs abstract information data such as sound and multimedia. Therefore, the use of big data analysis technology can effectively improve the effectiveness and comprehensiveness of university teachers’ corpus in the course of lesson preparation. The technical contributions of this article can be summarized as follows:

First, it constructs the multimedia integrated English teaching framework, and analyzes the hardware system framework and software system architecture. In addition, big data technology is used to screen and analyze the corpus.

Second, in order to solve the problem that the traditional recommendation algorithm ignores the time series of teachers and students’ browsing behavior, this article proposes a recurrent neural network model based on ant colony optimization. The convolutional neural network is used to mine the user’s potential preference characteristics, and the weighted hybrid recommendation strategy is used to get the hybrid recommendation model.

Third, we have carried out simulation experiments. The results show that the hybrid recommendation model has advantages in accuracy and recall rate, which indicates that the hybrid recommendation model can combine the advantages of the traditional recommendation model and the neural network recommendation model, and has certain advantages.

The rest of the article is organized as follows. The second part discusses the hardware implementation and software architecture of the multimedia fusion system based on big data technology. In the third part, an optimization algorithm based on an improved recurrent neural network is investigated. The fourth part presents the test results. Finally, the fifth part concludes the whole article.

## Related Work

Relevant scholars pointed out that the development of computer multimedia technology is a gradual accumulation process (Zheng, 2019). The developed multimedia technology is not only a comprehensive integration of the above technologies, but also a close combination of computer and image processing technology, thus forming a multimedia technology that takes the computer as the tool and the media such as collection, text, sound, moving and static images as the comprehensive processing object. The emergence of multimedia technology has formed a brand new technical content of computer and provided users with a brand new application environment (Kumar Chandar, 2019).

The research shows that multimedia information data management technology is based on database technology, but the traditional relational database deals with character and numerical data, which is not suitable for expressing the image, sound, video and other non-formalized multimedia data (Ponmalar & Valsalal, 2021). The current mainstream database system is mainly based on the original relational database extension, introducing a new binary large object (BLOB) data type in order to store multimedia object fields, but this proprietary data type will be completely or partially at the expense of portability (Li et al., 2022).

In order to improve the effectiveness and efficiency of patent translation, some scholars proposed a hybrid strategy based approach, that is, a combination of semantic analysis and rule-based translation techniques (Wu, 2021). This article uses semantic analysis technology to focus on the recognition of central verbs in sentences and the analysis of name phrases with nested structures in sentences. The semantic analysis results are input into a rule-based translation system, which has been integrated into the online Chinese-English machine translation system of the State Intellectual Property Office (Jin & Liu, 2010). However, it is undeniable that MT based on rules and semantic analysis requires engineers to enter the system in advance in order to find the prescribed “translation” for the source text (Klimova et al., 2023; Shi et al., 2023a; Kitchin, 2021). However, the amount of work involved in such entry is unimaginable. Before the arrival of more advanced AI translation, linguistics and related disciplinary and humanistic knowledge are exactly where human translation can make up, and translators will continue to play a large principal role in these aspects (Youssef, Eid & Agag, 2022).

Related scholars put forward a data classification rule extraction algorithm based on ant colony algorithm (Shi et al., 2023b). Based on the movement of ants and the division of data attributes, the precursors of corresponding rules are gradually formed through random search (Zhang, 2021). In the search method of ant colony algorithm, the rule construction method, pruning method and pheromone updating method are defined. The algorithm adopts the entropy measure method and combines it with pheromone updating to eliminate the error caused by the limitation of entropy measure (local heuristic measure). Compared with the typical classification algorithm CN2, the accuracy of this method is comparable to that of CN2, and the rule list found by this method is simpler than that obtained by CN2.

The researchers note that swarm intelligence appears in biological groups of some insect species (Bhat & Huang, 2021). It generates complex and often intelligent behavior through the interaction of many autonomous group members. Interaction is based on unsupervised primal instincts, and the result is the ability to perform very complex forms of social behavior and perform many optimization and other tasks. The main principle of these interactions is what is known as ”stigmergy forces,” or indirect forms of communication by changing the environment (Binsaeed et al., 2023). As the ant moves along the trail, it can sense this powerful hormone. It has an attractive effect on ants, and as a result, ants tend to follow paths with higher pheromone concentrations. This causes an autocatalytic reaction, or self-reinforcing positive feedback effect. Ants attracted to the pheromone release more of the same pheromone on the same path and cause more ants to be attracted. Swarm intelligence has many advantages due to the use of mobile agents and media. These properties make swarm intelligence very suitable for computer networks including ad-hoc wireless networks.

### The construction of a multimedia-integrated English teaching framework based on big data technology

#### The construction of a multimedia integration teaching framework

The construction of multimedia fusion mode of university English translation needs to reflect the function of teaching digitalization, intelligence and information service. Multimedia fusion technology platform is a teaching system installed on mobile devices, which can realize the transmission of teaching information resources in university colleges and universities through mobile devices, such as the multimedia fusion technology platforms with high application effect are micro classroom, intelligent classroom, cloud classroom, blue ink cloud learning class, flipped classroom, multimedia classroom, *etc* (Shi et al., 2022). Using a variety of modern information technology to develop and design a multimedia convergence network platform to provide personalized digital and intelligent services for teaching, change the way of learning experience of students and teaching experience of teachers, change the way of teaching and teaching means in universities and colleges, and then build a multimedia convergence teaching environment based on big data technology.

**Figure 1:**
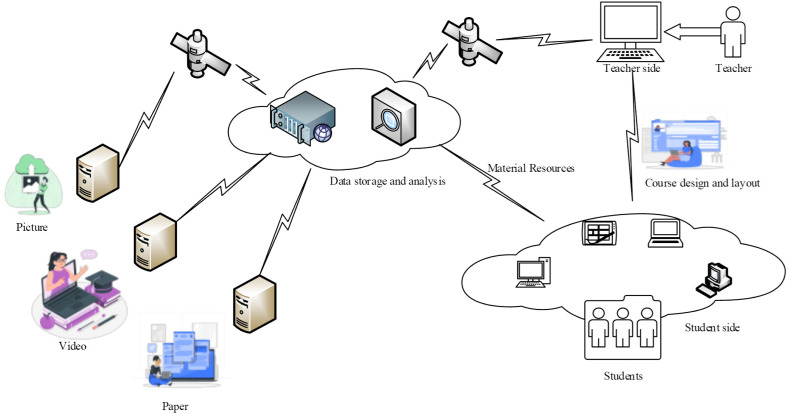
A system framework for multimedia convergence based on big data technology.

Its architecture is shown in Fig. 1. The server collects data university English translation-related materials and information as widely as possible by collecting images, videos, texts and other resources in databases and networks. These materials are aggregated into the cloud server through the network to form a multimedia library unique to the university English translation course (Mustafa et al., 2022). In this multimedia library, the big data screening program studies and organizes each of these materials, and organizes the characteristic information and tags of each material. When teachers use it, they design and organize the course according to their teaching style, and integrate and recommend the materials in the multimedia library through key words and descriptions. After the teacher designs the lesson, the layout and layout of the lesson and the corpus resources are aggregated in different ways to the local cloud where the students are located. Students can access the learning tasks and courseware in the local cloud through multiple terminals, which effectively improves students’ learning effectiveness.

When students finish their pre-study, they take a pre-study test on the multimedia integration platform. The platform will analyze and grade the students’ test intelligently according to the test standard answers uploaded by teachers in advance, and send it to teachers in the form of a learning analysis report. Teachers can clearly understand students’ pre-study situation on the multimedia integration platform, and then use it as a basis to make targeted teaching plans. Through the preparation before class, teachers can more accurately grasp the teaching objectives and teaching content, which will help the next multimedia integration activities. The students’ pre-study before class helps to develop their independent learning and intellectual abilities, and also helps to close the distance between teachers and students.

In the middle stage of the multimedia integration class, a model of teacher lecture, question and answer, student interaction, and post-class summary is used. Teachers use the teaching platform to organize discussion activities about the teaching content, issue classroom learning tasks, discuss key learning content in the form of group discussions, and send one person from each group to publish the discussion content, and teachers give praise to students according to group performance and publication. In this way, students’ creative thinking is stimulated. The teacher can easily select interactive sessions from the library of materials so that each student has the opportunity to perform in spoken English and stimulate students’ interest in learning through interactive communication methods.

Students can communicate and interact with their teachers and classmates after class through the multimedia integration platform, which breaks the previous time and space constraints and allows students to improve themselves at any time. Teachers can better understand students, can be targeted to individual counseling, according to the teaching situation of their own teaching to summarize and reflect, ready for the next course, so as to form a complete set of multimedia integration process.

**Figure 2:**
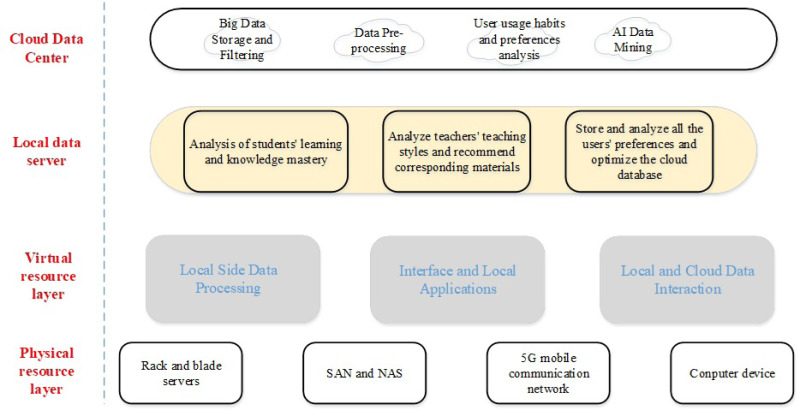
A software framework for multimedia convergence based on big data technology.

As shown in Fig. 2, the software architecture of English translation teaching based on multimedia integration of big data technology consists of four parts: cloud data center, local data server, virtual resource layer and physical resource layer. The bottom layer is the hardware devices applied by students and teachers, through which the terminal carriers of the software architecture are formed, such as computers and 5G cell phones. The third layer is some virtual interfaces, which are connected to the server, through which the data in the server is sent down to the user terminal in time, and the information of the user’s request is sent to the cloud for processing on the other hand. The second layer is the interaction layer for handling the user’s local data. It stores the data generated locally and interacts with the cloud data to ensure the stability of the terminal operation. The first layer is the core of the system, which processes and analyzes the large database in the cloud to provide fresh and accurate material for teachers. It also analyzes students’ preferences in the learning process and makes suggestions for teachers’ next teaching steps.

### A study of university English translation system based on multimedia integration technology

The development of multi-core computing technology brings more and more complex computing resources. Under this background, using a single operating system to manage system computing resources will lead to waste and unreasonable allocation of computing resources. Virtual computing technology provides the possibility to solve this problem, which subdivides a single system into multiple systems to accommodate more customer execution environments and more applications, thus improving the utilization rate of the system. Although virtual computing technology brings flexibility to high performance computing, different high performance computing applications have different requirements for computing resources. Therefore, it is particularly important to study a resource management system that can dynamically predict and adjust virtual computing resources according to application requirements. To solve the above problems, the computing resource management system for high-performance computing determines the behavior of applications in the virtual customer execution environment, especially the behavior of parallel programs commonly used in high-performance computing, and dynamically adjusts the computing resources in the execution environment to improve the performance and resource utilization of applications running in the virtual customer execution environment. At the same time, in the case of competing resource usage in multiple customer execution environments, resources can be dynamically configured based on the nature of the tasks running on the execution environment, thereby improving overall performance.

University English translation is a three-dimensional teaching system which is guided by teaching theory, applying the viewpoint and method of system science, reasonably selecting media information that meets the actual teaching according to the teaching objectives and the characteristics of teaching objects, and using computer technology to combine organically in the system to form an optimized teaching mode. Multimedia courseware should be based on combining the teaching objectives and tasks, exploring the reasonable and appropriate use of multimedia education technology, and establishing the practical training mode and evaluation mechanism of college English under the multimedia environment.

**Figure 3:**
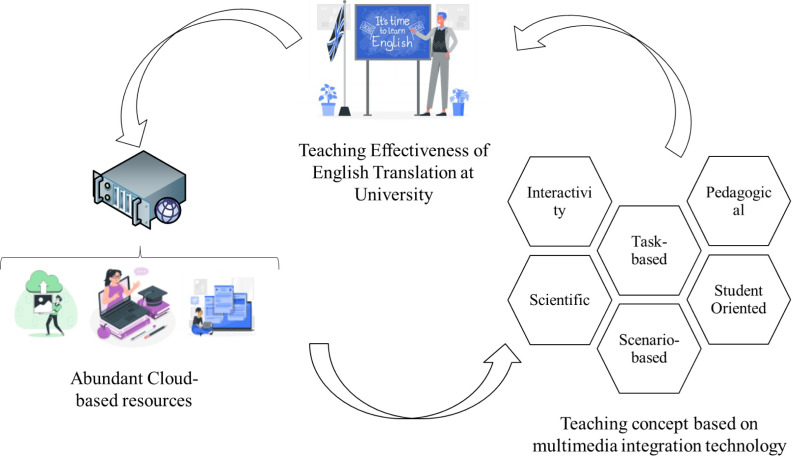
The concept of teaching English translation in university based on multimedia integration technology.

In Fig. 3 the teaching process of multimedia integration mode is divided into three parts: before, during and after class.

(1) In the pre-class preparation stage, teachers need to prepare for the teaching of the course, collect resources related to the teaching content on the Internet, and make the prepared materials into short videos, pre-study task sheets and test questions, *etc*., which are pushed to students through the multimedia integration platform and system, and students receive the materials on the platform to pre-study the learning content of the next class. In the pre-study process, if students have questions about the pre-study materials, they can send private messages to the teacher on the multimedia integration platform to communicate and exchange with the teacher.

(2) The in-class stage is the core part of the multimedia integration process, where teachers carry out the next teaching activities the pre-class teaching design. Avoiding the traditional teaching mode “one word” situation, to guide students to explore knowledge learning, group cooperative learning, focusing on highlighting the main position of students.

(3) After class, teachers design after-class homework according to students’ classroom situation and record micro-lesson videos, which are pushed to students through the multimedia integration platform, so that teachers can check students’ homework completion at any time. After the students finish the homework, the multimedia integration platform will summarize the wrong questions and put them in the wrong question bank so that students can practice again. Students independently analyze the mastery of classroom knowledge and can check the gaps through micro-lessons, and students make self-summary to improve their learning plan.

### Improved ant colony optimization algorithm based on improved recurrent neural network

#### Optimization algorithm based on improved recurrent neural network

Because of the fully interconnected structure of recurrent neural network, the network exhibits extremely complex dynamic behavior. As a dynamic system, various steady-state patterns are the basis for neural network system to simulate a series of intelligent activities such as learning, association, memory and pattern recognition of biological nervous system. Stability analysis is an important part of dynamic analysis of dynamic systems. Neural network has attracted wide attention because of its great theoretical and application value. For example, neural networks have been successfully applied in image and information processing, pattern recognition, and associative memory. It is well known that the various engineering applications of neural networks mentioned above depend critically on the stability and dynamic behavior of neural networks.

##### Long short term memory (LSTM) model construction.

The deep averaging network (DAN) model uses user and material content interaction behavior to capture similarities between users and items. The model focuses only on implicit feedback, *i.e.,* whether they have read a given article and in what order they viewed it. The order in which a user reads an article encapsulates information about the user’s interests, and the DAN model uses LSTM to learn the order dependencies of the articles read to capture the user’s interests (Shen et al., 2022). Users’ interests change dynamically over time, so a bidirectional LSTM structure is used. We select each user’s specific browsing history data as the input to the LSTM. A neural attention mechanism is also introduced to capture the similarity between users and items. Then, in order to learn the parameters of the model, the DAN model uses an objective function based on ranking learning. Finally, material is recommended to the user based on the similarity of the computed inner product vector between the user and the item.

DAN is designed as a parallel convolutional neural network (PCNN) consisting of two convolutional neural networks (CNN). The PCNN fuses the feature information of the material. DAN introduces an artificial neural network and designs an ARNN component to capture the historical readings of the user with potential sequential features (Henderson & Corry, 2021). ARNN is an attention-based recurrent neural network can obtain richer sequential features at different click times. An attention mechanism can be used to capture the different effects of user clicks on the candidate material to model the current interest of the user. The model structure is described in detail below.

Suppose the user’s historical click material is {*X*_*n*−*t*_, *X*_*n*−*t*+1_, …, *x*_*n*_}. where *x*_*n*_ is the nth clip clicked by the user. Given the candidate material as *x*_*n*_, the recommendation system takes {*X*_*n*−*t*_, *X*_*n*−*t*+1_, *K*, …, *x*_*n*_} as input and needs to calculate the probability of the user clicking on the candidate clip *x*_*n*_.

(1) \begin{eqnarray*}{X}_{t}= \frac{1}{n} \sum _{i=1}^{m}{V}_{i}{C}_{t}.\end{eqnarray*}

*V*_*i*_ represents the click speed, *X*_*t*_ represents the specific user, and *m* represents the number of user clicks. In the input gate, which indicates the information to be updated, the *i*_*t*_ values range from 0 to 1 and are used to select the proportion of new information to be remembered, and *C*_*t*_, which indicates the alternative cell state (Rodriguez-Medina, Dominguez-Isidro & Ramirez-Martinell, 2022).

(2) \begin{eqnarray*}{i}_{t}= \left( \sigma \left( {W}_{i}\cdot \left[ {h}_{t-1},{x}_{t} \right] \right) \right) +{b}_{i}\end{eqnarray*}

(3) \begin{eqnarray*}{C}_{t}=\tanh \nolimits \left( {W}_{c}\cdot \left[ {h}_{t-1},{x}_{t} \right] \right) +{b}_{c}.\end{eqnarray*}

The LSTM output results using sigmoid as the activation function, where the resulting user vector formula is shown in Eq. (4).

(4) \begin{eqnarray*}{y}_{user}=sig~\mathrm{mo}~id \left( w\cdot \left( {y}_{t}+{y}_{feature} \right) \right) = \frac{1}{1+{e}^{ \left( w\cdot \left( {y}_{t}+{y}_{feature} \right) \right) }} .\end{eqnarray*}

In Eq. (3), *w* represents the weights; *y* represents the output value; and dot product represents the convolution operation.

##### CNN model construction.

The input of the CNN is the word embedding vector of the material, denoted as {*X*_1_, *X*_2_, *K*…, *x*_*n*_}, and *U*_*I*_ denotes the result of the 1st convolution (Zhao et al., 2021).

(5) \begin{eqnarray*}{U}_{I}=\sum _{i=I}^{I+M}{X}_{i}\otimes W\end{eqnarray*}

Use global average pooling layer for dimensionality reduction:

(6) \begin{eqnarray*}{y}_{poll}= \frac{1}{m} \sum _{i=1}^{m}{U}_{i}.\end{eqnarray*}

Using the Sigmoid activation function, obtain the material text vector:

(7) \begin{eqnarray*}{y}_{material}=sig~\mathrm{mo}~id \left( {y}_{poll} \right) = \frac{1}{1+{e}^{ \left( {y}_{poll} \right) }} .\end{eqnarray*}

Obtain the user prediction score with the formula:

(8) \begin{eqnarray*}{y}_{pred}={u}_{i}{v}_{j}^{T}=\sum _{k=1}^{K}{u}_{ik}{v}_{kj}.\end{eqnarray*}

where *u* represents the user vector and *v* represents the item vector.

#### Ant colony algorithm based on improved recurrent neural network algorithm

Machine learning, as one of the important technical means in the era of artificial intelligence and big data, has been vigorously developed between the last two decades. Both academia and industry have applied machine learning technology to all aspects of life, such as daily video recommendation, headline recommendation, Meituan optimal path problem recommendation, drop optimal path problem recommendation and other application problems will use machine learning algorithms to do the corresponding application problems. For traditional machine learning, traditional machine learning, due to its strong theoretical nature, in which there are a large number of learning strategies, tends to have a better result in solving practical problems. The main process steps of traditional machine learning are data collection, feature engineering, building regression/classification prediction models, model accuracy evaluation and the final obtained regression/classification results. Therefore, this article proposes an Ant Colony Optimization algorithm with Hybrid Greedy Strategy (HSACO), which addresses the problem that random forests are highly influenced by parameters in solving application problems. The Ant Colony Optimization algorithm is used to optimize the hyperparameters and change the evolutionary direction of the population in the iterative process through the greedy strategy to accelerate the convergence speed of the Ant Colony Optimization method. Thus, the prediction accuracy of this algorithm in solving applications is improved.

For regression problems, redundant features waste many computational resources, thus the algorithm presents a high computational complexity. To improve the effectiveness of the algorithm for regression prediction, the data collection work is separated from the data pre-processing work, and the feature importance score is included as a separate step, from which the feature importance score can tell which features will produce greater results for the prediction results, and thus determine the reliability of the features. The algorithm flow is shown in the following figure.

**Figure 4:**
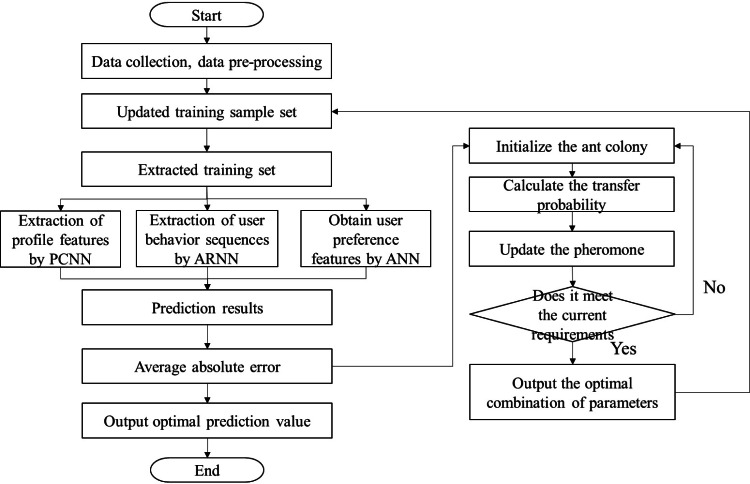
Recurrent neural network algorithm flow based on ant colony optimization algorithm.

As shown in Fig. 4, the algorithm flow starts with collecting and pre-processing the content of the material in the database. Then training and updating are performed according to the set parameters and data values. Then the profile features are extracted by PCNN, the user behavior sequences are extracted using ARNN and the user preference features are obtained using ANN to obtain the descriptive features of the dataset and the preference features of the current users. Predictions are made based on these features and prediction results are obtained. The gap between the current prediction parameters and the target parameters is obtained according to the test results. The parameters are optimized. Initialize the ant colony parameters, followed by calculating the transfer probability, determining whether the current error meets the requirements, and updating the pheromone. The current combination of parameters is output when the requirements are satisfied.

### Analysis of test results

#### Experimental environment and simulation settings

After building the recommendation system, the recommendation effect and performance need to be evaluated accordingly. The evaluation indexes mainly include accuracy index and non-accuracy index, among which accuracy index is the target of recommendation algorithm optimization, measuring the strength of the algorithm. The non-accuracy index is mainly to measure the goodness of the recommendation results, including the diversity, novelty and personalization of the recommendation results. This experiment evaluates the metrics based on precision (P), recall (R), F1 score (F), AUC, and weekly per capita click-through rate, weekly per capita exposure rate, and next-day retention rate. Each experiment was run 10 times, and 100 users were randomly selected in each execution. In order to ensure the effectiveness of the test, the experiment was conducted in A/B-test mode, in which two scenarios were developed for the same target, and one scenario was used randomly by groups of users with the same (similar) composition, and the user experience data and business data of each group were collected, and the best version was finally evaluated and formally adopted based on significance test analysis.

#### Verification of the superiority of the algorithm

The core recommendation idea of the improved RNN-based optimization algorithm is to find users who have similar preferences as the user, and then recommend to that user items that have been purchased by other users but have not been purchased by that user. However, both algorithms ignore the problem of the user’s chronological order in browsing news. To verify the effectiveness of the improved RNN-based optimization scheme, the method is compared with other commonly used ALS-based, content-based, and improved RNN-based algorithms.

**Figure 5:**
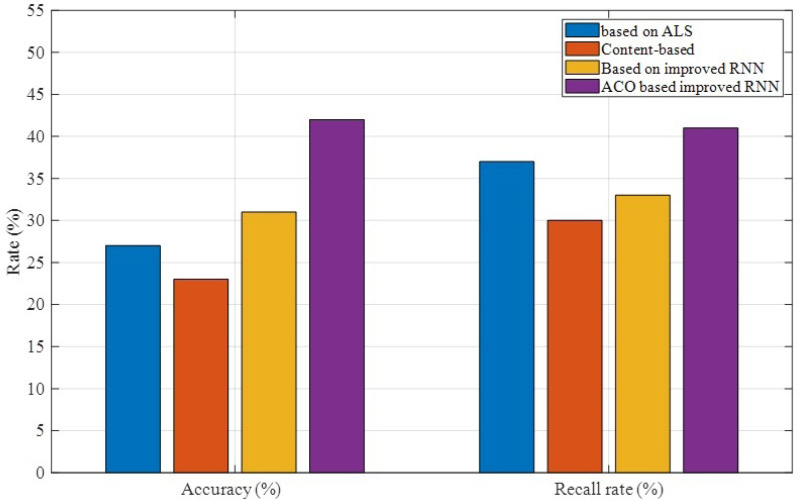
Accuracy and recall rate results comparison.

As shown in Fig. 5, in terms of accuracy, the improved optimization algorithm can better improve the accuracy of material prediction, that is, it can better extract the user’s preference features and the feature data of the material itself. And among the remaining three schemes, the improved RNN-based scheme performs better, which can show that the recurrent neural network algorithm has better adaptability in the field of optimal recommendation. In terms of recall rate, the proposed method is slightly better than the ALS-based algorithm. The recall rate of the proposed algorithm exceeds 40%. The recall rate of the improved RNN-based algorithm is relatively low, and it can be seen that new method overcomes this drawback to a certain extent.

**Figure 6:**
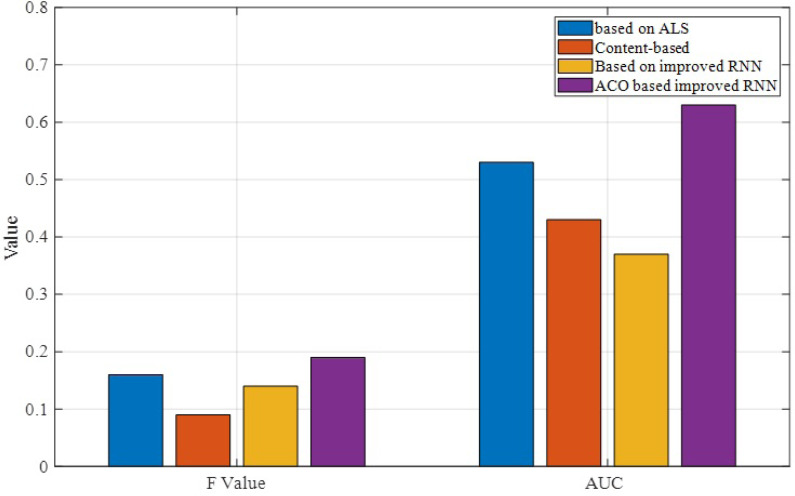
Comparison of F and AUC results.

As shown in Fig. 6, the F-value of the proposed algorithm is slightly higher than the ALS and IRNN algorithms, indicating the practical relevance of this algorithm in real applications. Therefore, a larger value of AUC means that the algorithm is more effective. From the above figure, we can see that the algorithm has the highest AUC value, while the ALS-based algorithm is slightly less effective.

**Figure 7:**
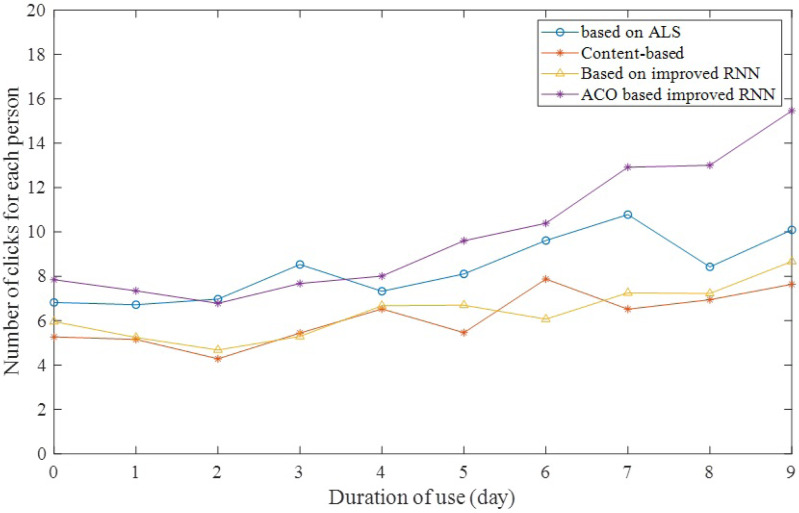
Number of clicks for each person.

The per capita click-through rate is a measure of how much users like the system itself as their usage time gradually increases. If the click-through rate per capita gradually increases with usage time, it indicates that the system itself is attractive. Users spend more time on the system per unit of time and are more interested in the recommended content in the system. As shown in Fig. 7, in the experiment, the click rate per capita increases significantly with the gradual increase of the user’s usage time, while the click rate of other methods increases slowly. This indicates that new scheme takes into account the influence of user click order and effectively improves the accuracy of the recommended material in the system.

**Figure 8:**
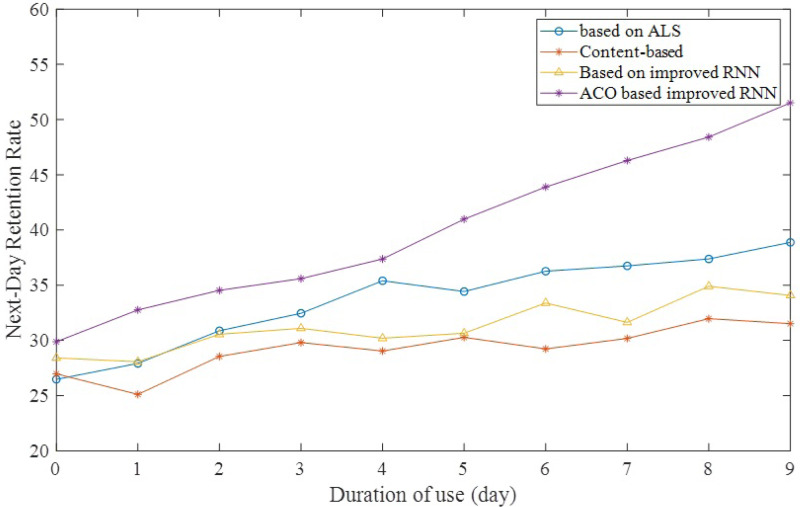
Next-day retention rate over time.

Next-day retention rate refers to the probability that a user will restart the program the next day after using it. Next day retention is an indexing of the quality of the market launch, *i.e.,* the next day retention the better the quality of the user can be understood. It is also a measure of user recognition of the value of the product. Figure 8 shows that the next-day retention rate increases gradually over time after adopting the new way to recommend material. This indicates that as the product is used, the recommendation algorithm learns more and more about the preferences of students and teachers, thus recommending better quality and more relevant materials.

### Ease of use analysis of the system framework

**Figure 9:**
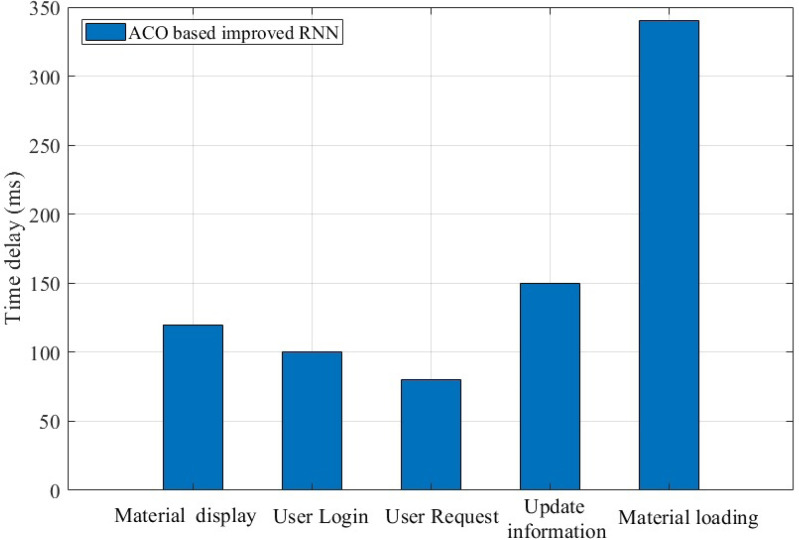
Response latency of the platform in handling various requests.

The above analysis shows that the algorithm proposed has certain advantages in terms of accuracy, next-day retention rate and effectiveness. In practice, the ease of use of the system itself is also a major aspect that affects the experience of teachers and students. From Fig. 9, we can see that the delay time of user login, user request and update information is mostly within 150 ms during the normal use of the system. The high delay of material loading is mainly due to the fact that the newly requested material needs to obtain data through the cloud.

## Conclusion

This article first investigates the development and connection between the state of development of the teaching model of multimedia integration. A framework for teaching English with multimedia integration was built. The components of the hardware system framework and software system architecture was analyzed. For the problem that the English corpus in the process of university English translation teaching was numerous and updated at length, the corpus was screened and analyzed using big data technology. To address the problem that traditional recommendation algorithms ignore the temporal sequence of teachers’ and students’ browsing behaviors, a recurrent neural network algorithm based on ant colony optimization algorithm was proposed, using recurrent neural network to model users’ time-series behaviors and convolutional neural network to mine users’ potential preference features. Then, a weighted hybrid recommendation strategy was used to train the weights of the three algorithms by logistic regression to obtain the hybrid recommendation model. Finally, experimental analysis was performed. According to the experimental data, the hybrid recommendation model performed better than the ALS-based recommendation model, the collaborative filtering-based model, and the improved RNN-based model in terms of precision and recall rates. This indicates that the hybrid recommendation model can combine the advantages of traditional recommendation models and neural network-based recommendation models, and has certain advantages in the application scenario of material recommendation.

 Computer networking is a complex dynamic distributed nonlinear system, network traffic analysis and prediction is a complex nonlinear problem. Modeling complex systems is still an open problem. Although some results have been achieved, there is not yet a widely applicable method for describing and predicting models, and there is still a lot of work to be done on how to use new technologies to model complex systems. In addition, the future prediction of the dynamic model of system theory is a fan gray area, which can only be accurate for the near-term prediction, and can only know a probability distribution for the long-term prediction. The method proposed in this article was only implemented in the simulation environment, and it needs to be perfected to adapt to the actual multimedia network college English translation environment represented by multimedia.

## Supplemental Information

10.7717/peerj-cs.1608/supp-1 Supplemental Information 1 Simulation code

## Additional Information and Declarations

### Data Availability

The following information was supplied regarding data availability: The code is available in the Supplemental Files.
